# The self-renewal dental pulp stem cell microtissues challenged by a toxic dental monomer

**DOI:** 10.1042/BSR20200210

**Published:** 2020-06-18

**Authors:** Gili Kaufman, Naima Makena Kiburi, Drago Skrtic

**Affiliations:** American Dental Association Science and Research Institute, Hagerstown, MD 21742, U.S.A.

**Keywords:** cytotoxicity, dental pulp stem cells, extracellular matrix, mineralization, self-renewal, TEGDMA

## Abstract

Dental pulp stem cells (DPSCs) regenerate injured/diseased pulp tissue and deposit tertiary dentin. DPSCs stress response can be activated by exposing cells to the monomer triethyleneglycol dimethacrylate (TEGDMA) and inducing the DNA-damage inducible transcript 4 (DDIT4) protein expression. The goal of the present study was to determine the impact of TEGDMA on the ability of DPSCs to maintain their self-renewal capabilities, develop and preserve their 3D structures and deposit the mineral. Human primary and immortalized DPSCs were cultured in extracellular matrix/basement membrane (ECM/BM) to support stemness and to create multicellular interacting layers (microtissues). The microtissues were exposed to the toxic concentrations of TEGDMA (0.5 and 1.5 mmol/l). The DPSCs spatial architecture was assessed by confocal microscopy. Mineral deposition was detected by alizarin red staining and visualized by stereoscopy. Cellular self-renewal transcription factor SOX2 was determined by immunocytochemistry. The microtissue thicknesses/vertical growth, surface area of the mineralizing microtissues, the percentage of area covered by the deposited mineral, and the fluorescence intensity of the immunostained cells were quantified ImageJ. DDIT4 expression was determined by a single molecule RNA-FISH technique and the cell phenotype was determined morphologically. DDIT4 expression was correlated with the cytotoxic phenotype. TEGDMA affected the structures of developing and mature microtissues. It inhibited the deposition of the mineral in the matrix while not affecting the SOX2 expression. Our data demonstrate that DPSCs retained their self-renewal capacity although their other functions were impeded. Since the DPSCs pool remained preserved, properties effected by the irritant should be restored by a proper rescue therapy.

## Introduction

Stem cells maintain their stemness in a process named self-renewal and differentiate into specialized ones [1]. Mesenchymal stem cells (MSCs) can heal and regenerate injured tissues due to their multipotent differentiation capacities, and immunomodulatory, angiogenic and neurogenic properties [2]. They can be isolated from a wide range of tissues including the dental pulp by expressing the mesenchymal stem cell markers CD105. Low oxygen environment is a critical factor that can affect their therapeutic values [3]. Dental pulp stem cells (DPSCs) express pluripotent/stem cell markers such as the transcription factors; octamer-binding transcription factor 4 (OCT4) and sex determined region y-box 2 (SOX2) [4]. DPSCs have more potent immunosuppressive, neurogenic, angiogenic and healing activities than other MSCs and they are used in regenerative therapies such as dental pulp and bone tissues regeneration [5].

Dental pulp tissue is surrounded by dentin and receives oxygen only through the vasculature. Consequently, oxygen tension in dental pulp might be lower than in the other tissues. This condition, in response to the pathological stimulation, induces a defensive reaction of dental pulp to maintain the stability of the internal environment [6]. Local hypoxic microenvironment can be a consequence of a concurrent oral inflammation, infection and/or injury [7]. Continuous cellular hypoxia can lead into oxidative stress and the generation of reactive oxygen species (ROS) that may cause damages to lipids, proteins and DNA and cause to an imbalance of redox homeostasis [8].

Triethyleneglycol dimethacrylate (TEGDMA), widely used co-monomer in restorative dentistry, exhibits adverse effects on cellular metabolism [9]. Commonly used as a diluent monomer in resin-based dental composites, the unpolymerized TEGDMA can easily leach out from the restorations, interact with the dental pulp tissue and cells. TEGDMA penetrates into the cytosol through the membrane of mammalian cells with the capability to negatively influence cell growth and development [10–13] and increases the level of ROS in the tissue [10,14].

The cellular damage by TEGDMA is caused by a coordinated induction of genes coding for biological processes. One of the most up-regulated genes related to the cellular stress response is DNA-damage inducible transcript 4 (DDIT4; [15]). DDIT4 is expressed at low levels in tissues and it is induced by cellular stresses such as hypoxia [16,17]. DDIT4 expressed under stress, turns off the metabolic activity triggered by the mammalian target of rapamycin (mTOR) signaling [17,18]. mTOR promotes the proliferation and differentiation of cells under adequate nutritional and environmental conditions and its activation is dependent on two complexes named mTOR complex1 and 2 (mTORC1 and mTORC2, respectively) [19,20]. mTORC1 controls proteins synthesis and cell survival through the phosphorylation of specific substances [21,22].

MSCs are triggered by their *in vivo* microenvironment including the extracellular matrix (ECM) which provide to the cells a complex array of physical signals including cell-cell contacts and cell-matrix adhesions, and biochemical signals which determine their spatial organization [23]. Self-renewal and lineage development is influenced by biochemical and biophysical cues provided by the reciprocal interactions between cells and ECM [1]. 3D microaggregate study indicates that higher cell densities induce self-renewal and maintain pluripotency [24]. Human MSCs cultured on soft hydrogels of stiffness comparable to the bone marrow self-renew and maintain stemness better than cells cultured on more rigid substrates such as polystyrene [25]. The initial tooth development is regulated by ECM such as basement membrane (BM). The matrix controls the polarity, proliferation and attachment as well as tooth size and morphology [26,27]. Later, dental pulp progenitor cells attached to the BM start differentiating into mesenchymal-derived odontoblasts that remain attached to the BM while being generated. Then, the BM discontinues and disappears at the initial stage of dentin mineralization [28,29]. Although ECM proteins are not considered a dentin-ECM proteins, the ECM components such as laminin reportedly promote odontoblast differentiation by inducing dentin sialoprotein synthesis while fibronectin is involved in the polarization of odontoblasts [30,31].

Collectively, the reconstruction of a new tissue, deposition of the tertiary dentin and maintenance of the stem cells reservoir are related to the DPSCs functions [32–34]. The present study was designed to determine the impact of the stress signaling on the healing potential and functionality of both human immortalized and primary dental pulp stem cells (hiDPSCs and hpDPSCs, respectively) upon exposure to the toxic concentrations of TEGDMA and activation of the DDIT4 pathway. hiDPSCs, considered less sensitive and reproducible, were used to validate the primary impact of TEGDMA. hpDPSCs, isolated directly from the pulp and preserving the original characteristics and functions of the tissue, provided a more relevant and reflective observation of the *in vivo* environment. We employed a 3D ECM/BM platform to mimic the native physiological and biochemical conditions of the microenvironment. Understanding the effect of stress on the functionality of DPSCs will help to determine if it is feasible to rescue and heal pulp tissue injured by leachables from dental restoratives and identify targets for a proper and effective treatment.

## Materials and methods

### Cell culturing

hpDPSCs were isolated from pulp tissues removed from third molars. The molars were obtained from healthy patients (aged 20–40 years) undergoing extraction at the NIH Dental Clinic and an informed consent was collected according to the guidelines. The study was reviewed and approved by American Dental Association (ADA) Institutional Review Board and was not considered to be human subject research. The teeth were broken into pieces in a sterile environment, exposing the pulp tissue in the pulp chamber and root canals. The pulp was gently isolated by using sterile tweezers. The excised pulp tissue was incised into 1–2 mm^2^ pieces and incubated in T-25 flask with DPSC BulletKit™ Medium (Lonza, Walkersville, MD, U.S.A.). Medium was replenished every 2–3 days. Cells outgrown from the pulp tissue explants [35] were collected after 14–21 days. hiDPSCs (TP-023 [36] were obtained as a gift from Dr. Lawrence T. Reiter, The University of Tennessee Health Science Center (UTHSC), Memphis, TN, U.S.A.) and approved by the UTHSC Institutional Review Board. An informed consent was obtained according to the guidelines. Both DPSCs were cultured in Dulbecco’s Modified Eagle Medium (DMEM; Invitrogen, Carlsbad, CA, U.S.A.). Media were supplemented with 10% fetal bovine serum, 2 mmol/l l-glutamine and 100 units/ml of penicillin and 100 μg/ml of streptomycin (Invitrogen, Carlsbad, CA, U.S.A.). At approximately 80% confluence, cell cultures were split at 1:5. All incubations were performed in a 5% CO_2_ humidified atmosphere at 37°C. TEGDMA (Esstech, Essington, PA, U.S.A.) was dissolved in ethanol to a stock concentration of 1 mol/l. Before utilization, a fresh 20 mmol/l TEGDMA batch was prepared by diluting the stock solution with the DMEM. TEGDMA solution was then sequentially diluted to the final concentrations of 0.5, 1.5, and 2.5 mmol/l. For 2D cell cultures, 1.7 × 10^4^ cells were seeded per well in 48-well plate (Greiner bio-one, Monroe, NC, U.S.A.).

### 3D ECM cultures

3D cell cultures were prepared by coating 48-well plate or Lab-Tek II Chambered Coverglass (8-well, Nalgene Nunc International, Rochester, NY, U.S.A.) surfaces with 100 μl of Matrigel™-ECM (ECM/BM; Corning, Tewksbury, MA, U.S.A.) and incubating for 30 min at 37°C. 1.7 × 10^4^ cells/well were seeded on the solidified ECM/BM and then incubated for 2 h. The adherent cells were covered with 100 μl of medium containing 10% ECM/BM. After 24 h of incubation, media was replaced with a fresh media containing the soluble monomer TEGDMA at different concentrations. Medium without the monomer served as a control. Cells were also seeded on coated wells/chambers according to the manufacturer instructions (2.5D cultures) and on uncoated surfaces and covered with similar volume of medium (2D control culture). The culture medium including TEGDMA was replenished twice weekly. For mature microtissues, TEGDMA was added after the microtissues were developed for 14 days.

### Single molecule RNA FISH

Single-molecule (sm) RNA FISH protocol was performed according to the manufacturer instructions. Cell monolayers on 2.5D cultures were fixed and permeabilized with 70% ethanol before the analysis. Cells were stained with Stellaris® FISH oligonucleotide probes for glyceraldehyde 3-phosphate dehydrogenase (GAPDH) labeled with Quasar® 570 dye and DDIT4 labeled with CAL Fluor® Red 610 dye (Stellaris oligonucleotides, Biosearch Technologies, Pataluma, CA, U.S.A.). DDIT4 customized oligonucleotide probes used in the present study are listed in Supplementary Table S1. The samples were washed twice with saline sodium citrate buffer (SSC, Ambion, Austin, TX, U.S.A.) with 10% formamide (Sigma–Aldrich, St. Louis, MO, U.S.A.). Cells cultured in coverglass chambers were submerged in Dulbecco’s phosphate-buffered saline (DPBS; Invitrogen, Carlsbad, CA, U.S.A.) for imaging with the motorized inverted Eclipse Ti-E epifluorescence microscope equipped with ×100 Plan Apo objective and a CoolSNAP EZ Monochrome Camera and filter set specific for the fluorophore. Images in the fluorescence channel were taken as a series of optical z-sections (0.35 microns per section) using the NIS-Elements software version 3.0 (Nikon Instruments Inc., Melville, NY, U.S.A.). Cell boundaries of the collected images of RNA FISH samples were identified and RNA spots were counted and localized using custom scripts in Matlab as described by ref. [37].

### Cell staining

2D and 3D cultures were washed twice with DPBS and fixed with 4% paraformaldehyde (Electron Microscopy Sciences, Hatfield, PA, U.S.A.). After permeabilization with 0.1% Triton X-100 (Alfa Aesar, Ward Hill, MA, U.S.A.) for 5 min, cell nuclei and F-actin were stained with 0.1 μM phalloidin and 1 μg/ml Hoechst for 30 min (Molecular Probes, Eugene, OR, U.S.A.), respectively. Cell viability was determined by fluorescence microscopy using LIVE/DEAD cell imaging kit (488/570, Molecular Probes, Eugene, Oregon, U.S.A.). Fluorescein and tetramethyl rhodamine optical filters were used to identify live and dead cells, respectively.

For immunostaining, cells were washed twice with 0.1% bovine serum albumin (Invitrogen, Carlsbad, CA, U.S.A.), blocked for 45 min with 10% normal donkey serum (GeneTex, Irvine, CA, U.S.A.), 0.3% Triton X-100 and 1% bovine serum albumin and then incubated with 10 μg/ml of anti-human endoglin/CD105 (MAB10971), GAPDH (MAB5718), SOX2 (MAB2018) and OCT4 (MAB1759) (R&D Systems, Minneapolis, MN, U.S.A.) monoclonal antibodies overnight at 4°C. Following the incubation, cells were washed twice and incubated with 1:200 dilution of donkey anti-mouse Immunoglobulin G (IgG; NL009, R&D Systems, Minneapolis, MN, U.S.A.) NorthernLights™ NL557-conjugated polyclonal antibodies, respectively, for an hour at 22°C, After washing and incubation with 14.3 mM 4′,6-diamidino-2-phenylindole (DAPI; Molecular Probes, Eugene, OR, U.S.A.) for 5 min at 22°C, the cells were washed again and kept in DPBS at 4°C before being analyzed.

### Mineralization

For detection of the mineral dentin secretion, DPSCs were cultured in a calcifying medium containing the standard media supplemented with 50 μg/ml ascorbic acid and 10 mM sodium β-glycerophosphate (Sigma–Aldrich, St. Louis, MO, U.S.A.) at 37°C for 14 days. The cells were fixed in 4% formaldehyde neutral solution and then stained with 1,2-dihydroxyanthraquinone (alizarin red S; Sigma–Aldrich, St. Louis, MO, U.S.A.) according to the manufacturer’s instructions and washed three time with distilled water by rotating for 1 h each at 22°C.

### Microscopy imaging and image analysis

Differential interference contrast (DIC) and fluorescence images of 2D cell cultures were taken under the Axio Vert.A1 inverted fluorescence microscope (Carl Zeiss; Jena, Germany) equipped with an AxioCam MRm CCD camera and a LED excitation light source (Thorlabs, Newton, NJ, U.S.A.) by the red fluorescent protein (RFP) and DAPI filters. The fluorescence intensity and length of individual cells, percentage of dead cells and viable cell density were counted and calculated by employing the Zeiss microscope software ZEN 2012 (blue edition) and ImageJ software (V.1.48, NIH).

The images of 3D cultures were collected by laser confocal scanning microscopy (LCSM; 3.5 mm intervals, two fluorescence channels; Leica Microsystems Inc., Buffalo Grove, IL, U.S.A.). To measure thickness and diameter of the layers, 25 constructs/microtissues were observed randomly per each experimental group. Thickness/vertical growth, size and fluorescence intensity of constructs was measured along both *x*–*z* and *y*–*z* planes by stacking images obtained by the LCSM and using the Leica Application Suite Advanced Fluorescence software (Leica Microsystems Inc., Buffalo Grove, IL, U.S.A.). The 3D structures obtained from the stacking images were analyzed by ImageJ software (V.1.48, NIH). The mineralized constructs were analyzed under stereomicroscope (Leica Microsystems Inc., Buffalo Grove, IL, U.S.A.) with two light sources at a constant magnification by using the Image-Pro software (Media Cybernetics, Rockville, MD, U.S.A.).

### Statistical analysis

The structural thicknesses/vertical growth and size of the 3D microtissues, number of mRNA molecules and cell length of individual cells were expressed as mean value ± one standard deviation of at least three separate experiments performed in triplicate. Statistical comparisons were performed using one-way analysis of variance (ANOVA) followed by the two-tailed Student’s *t*-test for the unpaired samples or one-way ANOVA followed by Tukey’s post-test for multiple comparisons. Results were considered statistically significant when *P*≤0.05. Statistical analysis of the constructs thickness and boxplots were computed by using the statistical software package “R” (version 3.2.1).

## Results

### TEGDMA effects the survival and growth of and induces DDIT4 expression in stressed DPSCs

**Figure 1:**
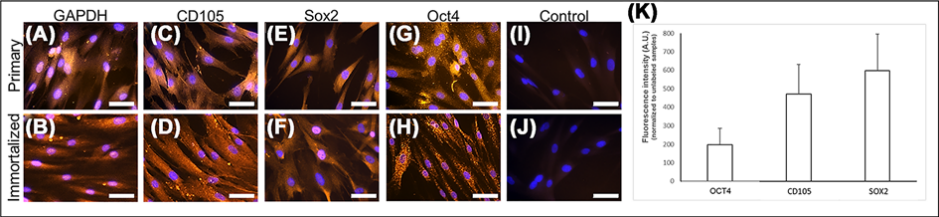
Human DPSCs expressing self-renewal capabilities hpDPSCs (**A,C,E,G,I**) and hiDPSCs (**B,D,F,H,J**) immuno-stained for GAPDH (**A,B**) CD105 (**C,D**), SOX2 (**E,F**), and OCT4 (**G,H**). Cells exposed to the secondary antibody only (negative controls: **I,J**). Blue – nuclei; orange – marker. Bar scale = 20 μm. Expression levels of OCT4, CD105, and SOX2 in hpDPSCs (**K**).

**Figure 2:**
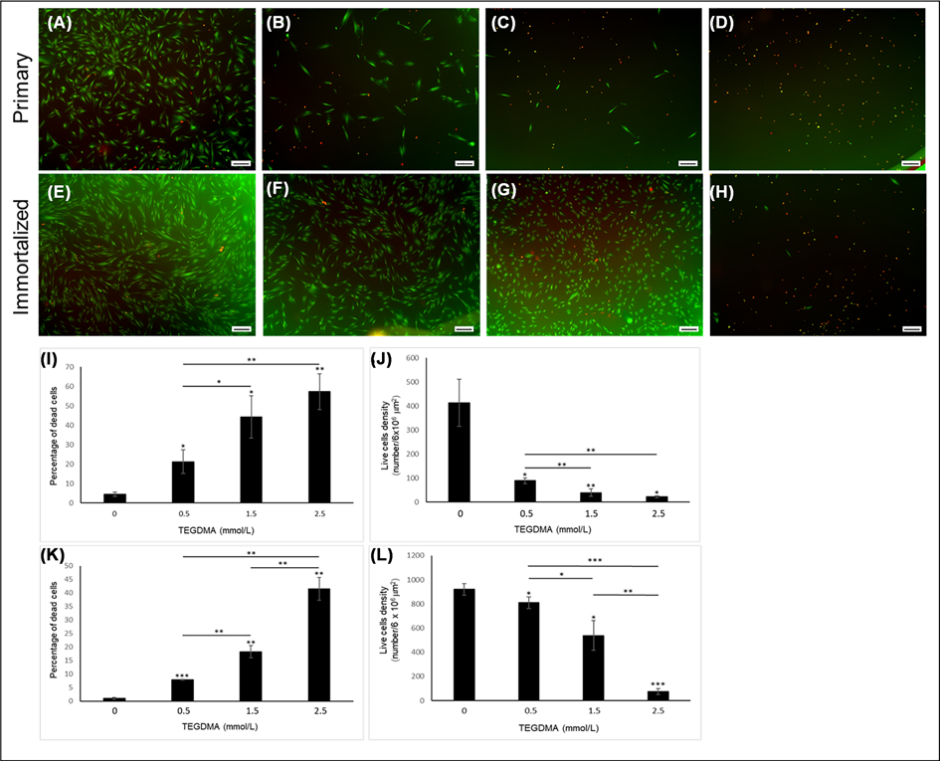
The viability and proliferation of hiDPSCs and hpDPSCs treated with increasing concentrations of TEGDMA LIVE/DEAD fluorescence images of hpDPSCs (**A–D**) and hiDPSCs (**E–H**) treated with 0 (**A,E**), 0.5 (**B,F**), 1.5 (**C,G**), and 2.5 (**D,H**) mmol/l. Green – live, red – dead. Bar scale = 200 μm. Bar graph representing hpDPSCs (**I,J**) and hiDPSCs (**K,L**) percentage of dead cells (**I,K**) and live cells density (**J,L**) exposed to increasing concentrations of TEGDMA. *P≤0.05, **P≤ 0.01, ***P≤ 0.001. Cells were incubated for 72 h.

hpDPSCs and hiDPSCs expressing the house keeping gene marker GAPDH (Figure 1A,B) expressed the mesenchymal marker CD105 (Figure 1C,D). The expression level of CD105 in hpDPSCs were 470.05 ± 160.62 arbitrary units (AU) (Figure 1K). Both cells expressed the self-renewal markers SOX2 (Figure 1E,F) and OCT4 (Figure 1G,H). The expression levels SOX2 and OCT4 in hpDPSCs were 598.27 ± 196.84 and 198.34 ± 89.51 AU, respectively. A low background was observed in cells immuno-stained only with the secondary antibody (negative control; Figure 1I,J). Increasing the concentration of TEGDMA decreased the viability of both hpDPSCs and hiDPSCs and changed their morphology from a spindle- to a round-shaped cells (Figure 2A–H, respectively). The percentages of dead cells increased significantly (p≤0.01) 2-, 3-, and 41-folds for hpDPSCs and 8-, 18-, and 41-folds (*P*≤0.001 and *P*≤0.01) for hiDPSCs (Figure 2I,K). Viable cell densities decreased by 324, 374, and 391 cells/6 × 10^6^ μm^2^ (*P*≤0.05 and *P*≤0.01) for hpDPSCs and 110, 282, and 746 cells/6 × 10^6^ μm^2^ (*P*≤0.05 and *P*≤0.001) for hiDPSCs when treated with 0.5, 1.5, and 2.5 mmol/l of TEGDMA compared with the non-treated cells, respectively (Figure 2J,L).

**Figure 3:**
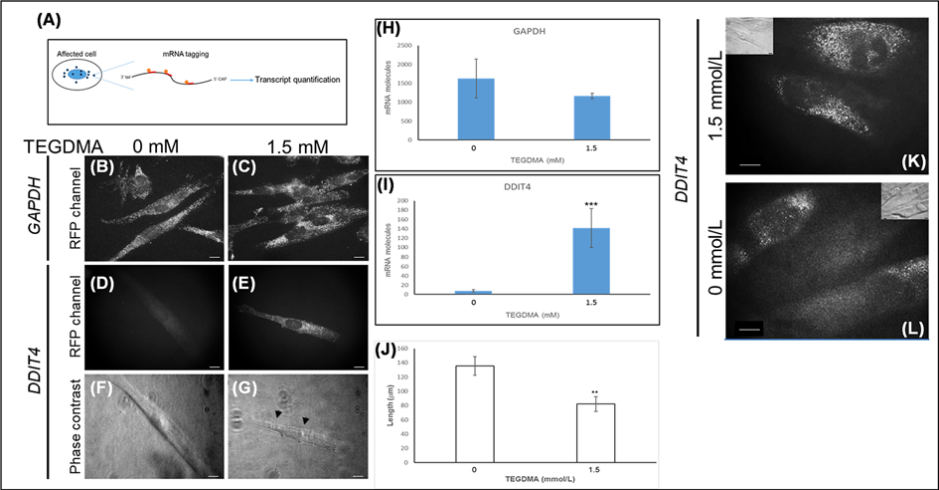
The expression of DDIT4 transcripts in human DPSCs exposed to TEGDMA on ECM substrate Schematic of the smRNA FISH technique (**A**). Expression of GAPDH (**B,C**) and DDIT4 (**D,E**) transcripts (white dots) in non-exposed hpDPSCs (**B,D,F**) and hpDPSCs exposed to 1.5 mmol/l TEGDMA (**C,E,G**). Phase contrast images of the non-treated (**F**) and treated (**G**) cells (arrows point to small vacuoles). The mRNA molecules of GAPDH (**H**) and DDIT4 (**I**) in treated and non-treated cells (**P≤0.01, ***P≤ 0.001). Comparison of the cell sizes of both samples (**J**). Fluorescent images of treated (**K**) and non-treated (**L**) hiDPSCs expressing DDIT4 transcripts with the corresponding phase-contrast images inserted. Bar scale = 10 μm.

The expression of DDIT4 gene facilitated by the single molecule RNA-FISH in cells affected by TEGDMA is shown in Figure 3A. GAPDH transcripts appeared in both non-treated and TEGDMA-treated hpDPSCs (Figure 3B,C). Difference between the number of GAPDH transcripts in the non-treated (1680 ± 253 mRNA molecules) and TEGDMA-treated cells (1130 ± 90 mRNA molecules) were not statistically significant (Figure 3H; *P*>0.05). Transcripts encoding for DDIT4 in the non-treated cells were barely observed (12 ± 3.42 mRNA molecules) compared with TEGDMA-treated (142 ± 45.24 mRNA molecules) counterparts (Figure 3D,E). This, more than 11-fold difference (Figure 3I) is of high statistical significance (*P*≤0.001). Cells expressing high levels of DDIT4 transcripts demonstrated the accumulation of vesicles (Figure 3F,G) and a significant (*P*≤0.01) size reduction (from 135.51 ± 13.04 to 82.24 ± 10.36 μm; Figure 3J). Similar trends in DDIT4 expression were observed in hiDPSCs treated with 1.5 mmol/l of TEGDMA vs. the non-treated group (Figure 3K,L).

### Stress prevents cell growth, interaction and development of microtissue, affects cell shape, and disassembles mature microtissue

**Figure 4:**
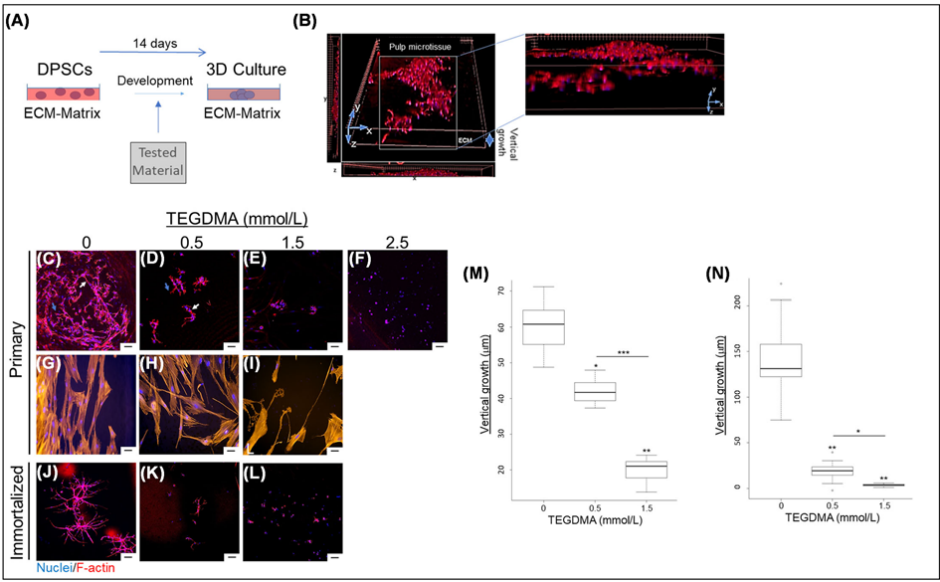
The spatial effect of TEGDMA on the structural development of human DPSCs microtissues Schematic of the experimental assay (**A**). 3D image of microtissue vertical growth (**B**). Confocal images of hpDPSCs (**C–I**) and hiDPSCs (**J–L**) microtissues developed in non-exposed cells (**C,G,J**) and cells exposed to 0.5 mmol/l TEGDMA (**D,H,K**), 1.5 mmol/l TEGDMA (**E,I,L**), and 2.5 mmol/l of TEGDMA (**F**). White arrows point to cell–cell interactions; blue arrows point to cell–ECM interactions. A comparison between 3D (**C–E**) and 2D (**G–I**) growth of primary cells. Vertical growth (thickness) of hpDPSCs microtissues (**M**) and hiDPSCs microtissues (**N**) as a function of TEGDMA concentration. *P≤0.05, **P≤0.01, ***P≤0.001. Bar scale = 50 μm.

Vertical growth, interaction and spatial development into microtissues of TEGDMA-exposed DPSCs is presented in Figure 4A,B. Increasing TEGDMA concentrations interfered with the growth and elongation of hpDPSCs, cell–cell interactions and the creation of spatial structures (Figure 4C). At 0.5 mmol/l of TEGDMA (Figure 4D), cell elongation, interactions with the adjacent cells (pointed by the white arrow) and nearby spheroidal structures (pointed by the blue arrow) were suppressed. At 1.5 mmol/l TEGDMA (Figure 4E) the suppressing effects on cell elongation and interactions intensified further. Cells were observed as aggregates. At 2.5 mmol/l TEGDMA, cell adhesion was prevented, and individual cells were observed (Figure 4F). Since the purpose of the study was to explore 3D structures, the 2.5 mmol/l TEGDMA treatment was excluded from further evaluations. The comparative assays in 2D cultures without ECM/BM, suggested that, at increased TEGDMA concentrations, the cytoplasmic volume decreased resulting in narrow and thin cell shape (Figure 4H,I). Similar TEGDMA concentration effect was seen in 3D hiDPSCs cultures in comparison with the non-treated cultures (Figure 4K,L vs. J). The impact of TEGDMA on the structural development of the microtissue was quantified as thickness (vertical growth). At concentrations of 0.5 and 1.5 mmol/l (Figure 4M), TEGDMA significantly decreased the thicknesses of hpDPSCs [(41.60 ± 3.51 μm; *P*≤0.05; 31% reduction) and (20.40 ± 2.66 μm; *P*≤0.01; 66% reduction), respectively] compared with the non-treated group (59.90 ± 7.30 μm). The difference between the two TEGDMA groups was highly significant (*P*≤0.001). A significant decrease (*P*≤0.01) in the vertical growth was also observed when the hiDPSCs were challenged by the same concentrations of TEGDMA. The reductions were 84% (22.93 ± 8.70 μm) and 97% (3.66 ± 1.1 μm), respectively, in comparison with the non-treated group (140.46 ± 32.90 μm). As in hpDPSCs group, the differences between the two TEGDMA-treated groups were significant (*P*≤0.05) (Figure 4N).

**Figure 5:**
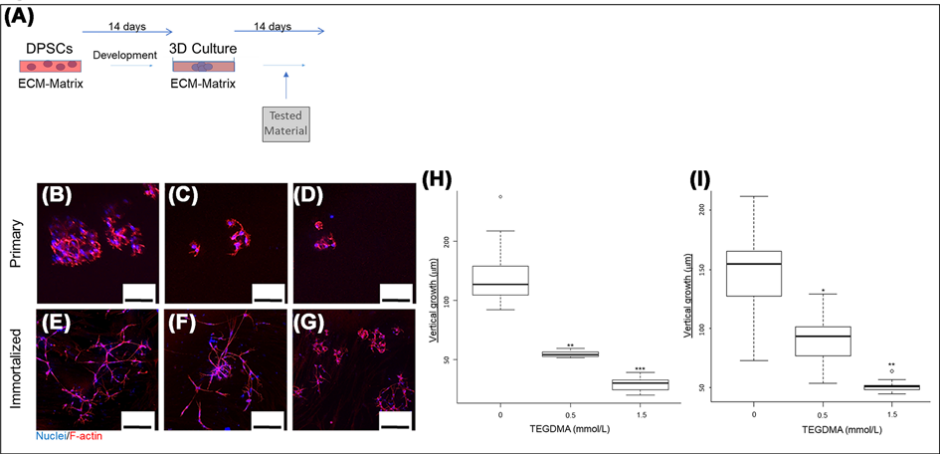
The spatial effect of TEGDMA on the structural deterioration of human DPSCs mature microtissues Schematic the experimental assay (**A**). Confocal images of the hpDPSCs (**B–D**) and hiDPSCs (**E–G**) mature microtissues of non-exposed cells (**B,E**) and cells exposed to 0.5 mmol/l TEGDMA (**C,F**) and 1.5 mmol/l of TEGDMA (**D,G**). White arrows point to cell–cell interactions and blue arrows point to cell–ECM interactions. Vertical growth (thickness) of hpDPSCs microtissues (**H**) and hiDPSCs microtissues (**I**) as a function of TEGDMA concentration (*P≤0.05, **P≤0.01, ***P≤0.001). Bar scale = 100 μm.

The impact of TEGDMA on mature hpDPSC microtissues was tested as illustrated in Figure 5A. At 0.5 mmol/l TEGDMA, the shortened length of the elongated cells led to a separation between the adjacent cells (Figure 5C). At 1.5 mmol/l TEGDMA, further shortening of cell length (Figure 5D) led to a point where they were barely observed in comparison with the structures of the non-treated microtissues (Figure 5B). Similar results were seen in hiDPSCs as well (Figure 5F,G vs. E). The decrease in the thicknesses of the microtissues correlated with their visual deteriorations follows. At 0.5 and 1.5 mmol/l of TEGDMA, the thicknesses of hpDPSC microtissues significantly decreased (55%; 54.55 ± 2.01 μm; *P*≤0.05 and 75%; 29.55 ± 5.22 μm; *P*≤0.01, respectively) compared with the non-treated samples (117.12 ± 21.17 μm). Differences between two TEGDMA-treated groups were significant (*P*≤0.05; Figure 5H). Similar trends were seen with hiDPSCs. Reductions in hiDPSC microtissue thickness were 43%, average thickness (89.73 ± 18.30 μm; *P*≤0.05) and 68%, average thickness (50.83 ± 4.76 μm; *P*≤0.01) for 0.5 and 1.5 mmol/l TEGDMA, respectively (Figure 5I).

### Stress interferes with the growth of the mineralizing microtissues and their capabilities to deposit mineral in the matrix

**Figure 6:**
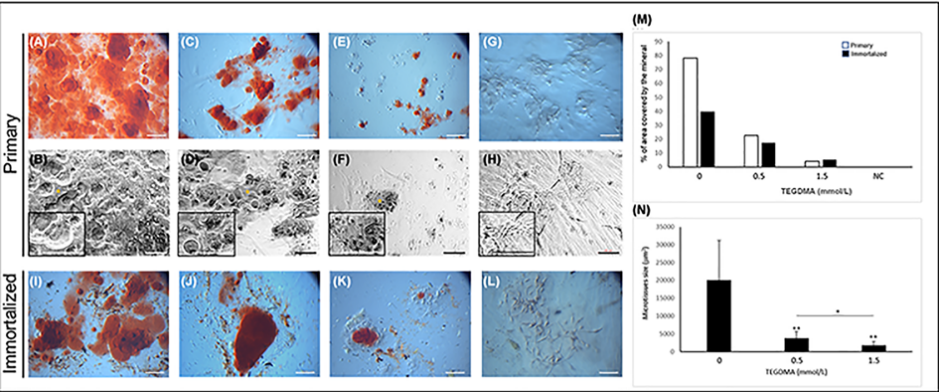
The impact of TEGDMA on the capability of human DPSCs microtissues to deposit the mineral in the matrix Stereomicroscopic (**A,C,E,G,I–L**) and phase-contrast (**B,D,F,H**) images of mineralization induced in non-treated cells (**A,B,I**) and cells treated with 0.5 mmol/l TEGDMA (**C,D,J**) and 1.5 mmol/l TEGDMA (**E,F,K**). Non-treated, non-mineralized microtissues serving as negative control, NC (**G,H**). Alizarin red S-stained calcium deposits are marked by asterisk. The percentage of area covered by deposited mineral (**M**) and the size of primary mineralizing microtissues (**N**) as a function of TEGDMA concentration (*P≤ 0.05, **P≤ 0.01). Bar scale = 100 μm.

Increasing the concentration of the monomer decreased the hpDPSCs capability to deposit the mineral in the matrix (Figure 6A–F), in comparison with non-mineralized/non-treated microtissues (Figure 6G,H). The percentage area of the matrix covered by mineral in hpDPSC microtissue cultures treated with 0.5 and 1.5 mmol/l of TEGDMA decreased 3- and 18-fold (average coverage 22.5% and 4.2%, respectively) compared with the non-treated cultures (average coverage 78%). In going from 0.5 to 1.5 mmol/l TEGDMA, the area of the mineral-covered microtissues decreased 5-fold. The decrease in the percentage of mineral coverage correlated well with the decrease in the surface area of the mineralizing hpDPSC microtissues. The latter decreased 5- and 11-fold [(3.8 ± 1.8) × 10^3^ μm^2^ and (1.8 ± 1.0) × 10^3^ μm^2^, respectively] compared with the non-treated control [(2.0 ± 1.0) × 10^4^ μm^2^]. Similar results were observed with the hiDPSC mineralizing microtissues (Figure 6I–L). In going from 0.5 to 1.5 mmol/l TEGDMA, the area of mineral-covered microtissues decreased 2-fold (*P*≤0.05; Figure 6N). The percentage area of the matrix covered by mineral in hiDPSCs decreased more than 2- and 5-fold (average coverage 17.39% and 5.27%, respectively) in comparison with the non-treated samples (average coverage 39.8%) (Figure 6M).

### Stressed cells continue expressing SOX2

**Figure 7:**
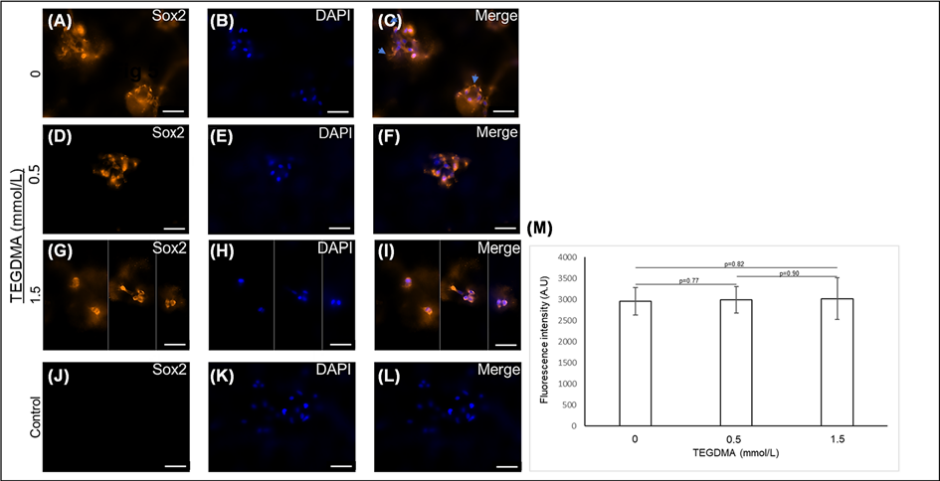
The impact of TEGDMA on the expression of the self-renewal marker SOX2 in human DPSCs microtissues Fluorescent images of developing hpDPSC microtissues of non-exposed cells (**A–C**) and cells exposed to 0.5 mmol/l TEGDMA (**D–F**) and 1.5 mmol/l TEGDMA (**G–I**). Cells exposed to the secondary antibody only; negative controls, NCs (**J–L**). Cells immune-stained for SOX2 (**A,D,G,J**) and nuclei (**B,E,H,K**) and the merged images (**C,F,I,L**). Fluorescence intensities normalized to the corresponding NC as a function of TEGDMA concentration (**M**). Blue arrows point to cells/ECM interactions. Bar scale = 50 μm.

The expression of the SOX2 marker in the developing microtissues was not affected by exposure to the increasing concentrations of TEGDMA (Figure 7D–I) in comparison with the non-treated microtissues (Figure 7A–C). The control group treated with a secondary antibody only showed a very low background (Figure 7J–L). However, the structural development of these microtissues was arrested as described before. The fluorescence intensity of the marker was similar among the samples treated with 0.5 and 1.5 mmol/l TEGDMA and the non-treated samples (2.99 ± 0.31) × 10^3^ AU and (3.01 ± 0.49) × 10^3^ AU vs. (2.96 ± 0.33) × 10^3^ AU, respectively, without any significant differences between the groups (*P*>0.05) (Figure 7M).

## Discussion

Dental pulp mesenchymal stem cells play a unique role in pulp tissue regeneration by maintaining the stemness of the cells on one hand and differentiating into odontoblast-like cells to create and deposit the tertiary (reparative) dentin on the other [3]. They maintain the stemness by the expression of core transcriptional factors associated with the stem cell proliferation/self-renewal markers OCT4 and SOX2 [38–40] and mesenchymal stem cell markers such as CD29, CD73, CD90, and CD105 [41,42], Tissue exposure to toxic monomers, such as TEGDMA, is known to induce stress response mechanisms in the cells and lead to apoptosis or tissue necrosis [12,43–45]. TEGDMA induces cell damage in both hpDPSCs and hiDPSCs by (i) causing a decrease in cell viability and proliferation as described previously for dental papilla cells [12] and (ii) a coordinated induction of genes coding for biological processes [46,47]. One of the most up-regulated genes was DDIT4. It is a major stress response protein involved in response to the cytotoxic impact of the monomer [15]. DDIT4 induction in stem cells was correlated to the incapability of hpDPSCs and hiDPSCs exposed to 1.5 mmol/l of TEGDMA to grow, elongate and develop while forming apoptotic vesicles on 2.5D ECM/BM matrix cultures. It has been previously shown [13] that TEGDMA, leaching from a composite with a distance of less than 1 mm, can reach pulp tissue in a concentration range of up to 4 mmol/l. Growth arrest was also demonstrated when both DPSCs in 3D matrix were exposed to the increasing concentration of TEGDMA. It prevented cell development and interactions with the adjacent spherical structures to create a mature microtissue. A parallel 2D observation indicated a morphological change in the volume of hpDPSCs. TEGDMA affected cell growth/elongation and, as its concentration increased, the interconnecting cells between the spherical aggregates vanished. The same mechanism was observed in mature microtissues exposed to increasing concentrations of TEGDMA. There, it led to cell size reduction, degradation of intercellular interactions and disassembly of the spatial structure. The reduction in the vertical growth of the developing microtissues and the thicknesses of the mature microtissues is inertly correlated to the structural alternations of the microtissues and the increasing concentration of the monomer. These results are consistent with our previously reported findings [12]. These modifications can be explained by the expression of DDIT4 and its inhibitory effect on the mammalian target of rapamycin complex 1 (mTORC1) signaling. Since mTORC1 controls protein synthesis and cell survival through the phosphorylation of the substrates S6K and 4E-BP1 [17,48], DDIT4 suppresses the activation of mTORC1 signaling by dephosphorylating both mTOR kinase substrates and, therefore, mTOR kinase activity. The suppression leads to a decrease in cell growth rate and a reduction of cell volume [49]. Recently, mTOR signaling was reported to control cell adhesion molecules such as E- and N-cadherins. The mTOR/cadherin signaling is required for cell adhesion and its inhibition may contribute to (i) inability of the interconnecting cells to interact and form the microtissue intact structures and (ii) cell–cell separation in mature microtissues when DDIT4 is activated [50,51]. Future efforts should focus on investigating the correlation between the activation of stress response protein DDIT4 pathway and the expression of adhesion molecules in DPSCs/ECM environment.

Stress response of hpDPSC and hiDPSC microtissues exposed to increasing concentration of TEGDMA decreased the size of the calcifying microtissues, and the portion of the mineral deposited in the matrix. These findings correlate well the observations indicating that the induction of DDIT4 expression inhibits the differentiation of mesenchymal stem cells while mTOR signaling promotes this process [18,52]. It is speculated that stem cells may repress mTOR activation in order to maintain a quiescent state and prevent an accelerated differentiation and depletion of their pool and function [18,53,54].

The present study demonstrated that TEGDMA-challenged DPSCs retain their stemness and self-renewal capacity by inducing the expression of the marker SOX2, a central regulator of pluripotency and stemness factors such as OCT4 [55,56], despite compromised development/maintenance of spatial structures and the ability to form dentin layers. Such outcome could be related to the multiple roles of DDIT4 in these processes. It has been shown [18] that DDIT4 regulates mTOR signaling and stemness of MSCs localized in hypoxic perivascular microenvironment by promoting the expression of OCT4 and SOX2 markers and suppressing spontaneous or excessive differentiation. It has also been reported that long-term inhibition of mTOR and the excessive differentiation prevent aging and maintain stemness of various stem cell types [52]. The impact on the capabilities of DPSCs to differentiate into multi cell lineages such as osteoblasts, adipocytes, chondroblasts neural cells [57] should be further explored and determined. Hypoxia caused by injury leads to excessive oxidative stress by the production of ROS. Bakopoulou [10] and Nocca [14], suggest that TEGDMA causes oxidative stress responses in dental pulp cells by providing a similar phenotype as hypoxia and the generation of ROS. DDIT4 helps retaining the quiescence of MSCs in hypoxic environment by inducing the expression of OCT4 and SOX2 [58]. Moreover, it has been revealed that expression of stem cell markers and self-renewal capabilities also elevates the viability of other cell populations by secreting stimulatory factors [59].

**Figure 8:**
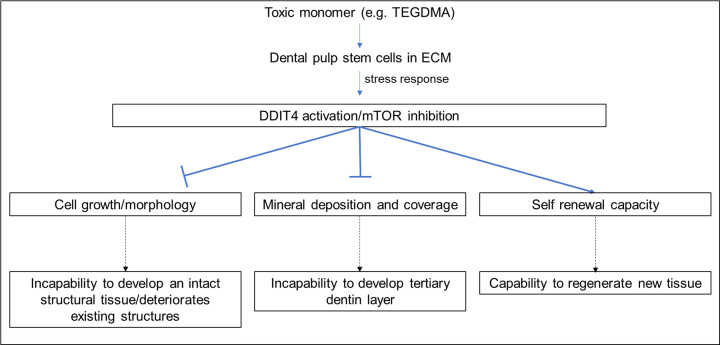
Schematic diagram summarizing the impact of TEGDMA on stem cell construction, mineral deposition, and stemness in ECM microenvironment

The capacity of DPSCs to maintain pluripotency/self-renewal markers such as SOX2 under TEGDMA challenge accentuates their potential to counteract toxicity, regenerate/replace the injured pulp tissues and mediate dentinogenesis to create a dentin pulp-like complex *in vivo* [60]. Consequently, a rescue treatment/therapy should be prudent to overcome the inhibitory effects of toxic materials on cellular reconstructive and healing capability (Figure 8).

## Supplementary Material

Supplementary Table S1

## Abbreviations

ANOVA: analysis of variance
AU: arbitrary units
BM: basement membrane
DAPI: 4′,6-diamidino-2-phenylindole
DDIT4: DNA damage inducible transcript 4
DIC: differential interference contrast
DMEM: Dulbecco’s modified Eagle medium
DPBS: Dulbecco’s phosphate-buffered saline
DPSCs: dental pulp stem cells
ECM: extracellular matrix
ECM/BM: extracellular matrix/basement membrane
FISH: fluorescence *in situ* hybridization
GAPDH: glyceraldehyde 3-phosphate dehydrogenase
hiDPSCs: human immortalized dental pulp stem cells
hpDPSCs: human primary dental pulp stem cells
hTERT: human telomerase reverse transcriptase
IgG: immunoglobulin G
LCSM: laser confocal scanning microscopy
MSC: mesenchymal stem cells
mTOR: mammalian target of rapamycin
mTORC1: mammalian target of rapamycin complex 1
NC: negative control
OCT4: octamer-binding transcription factor 4
RFP: red fluorescent protein
ROS: reactive oxygen species
SOX2: sex determined region y-box 2
TEGDMA: triethylene glycol dimethacrylate

## References

[1] Nava M.M., Raimondi M.T. and Pietrabissa R. (2012) Controlling self-renewal and differentiation of stem cells via mechanical cues. J. Biomed. Biotechnol. 2012, 797410 10.1155/2012/797410 23091358 PMC3471035

[2] Han Y., Li X., Zhang Y., Han Y., Chang F. and Ding J. (2019) Mesenchymal stem cells for regenerative medicine. Cells 8, 10.3390/cells8080886 PMC6721852 31412678

[3] Ledesma-Martinez E., Mendoza-Nunez V.M. and Santiago-Osorio E. (2016) Mesenchymal stem cells derived from dental pulp: a review. Stem Cells Int. 2016, 4709572 10.1155/2016/4709572 26779263 PMC4686712

[4] Liu L., Wei X., Ling J., Wu L. and Xiao Y. (2011) Expression pattern of Oct-4, Sox2, and c-Myc in the primary culture of human dental pulp derived cells. J. Endod. 37, 466–472 10.1016/j.joen.2010.12.012 21419292

[5] Yamada Y., Nakamura-Yamada S., Kusano K. and Baba S. (2019) Clinical potential and current progress of dental pulp stem cells for various systemic diseases in regenerative medicine: a concise review. Int. J. Mol. Sci. 20, 10.3390/ijms20051132 PMC6429131 30845639

[6] Wei F., Yang S., Xu H., Guo Q., Li Q., Hu L. et al. (2015) Expression and function of hypoxia inducible factor-1alpha and vascular endothelial growth factor in pulp tissue of teeth under orthodontic movement. Mediators Inflamm. 2015, 215761 10.1155/2015/215761 26441483 PMC4579319

[7] Li L., Zhu Y.Q., Jiang L., Peng W. and Ritchie H.H. (2011) Hypoxia promotes mineralization of human dental pulpcells. J. Endod. 37, 799–802 10.1016/j.joen.2011.02.028 21787492

[8] Nita M. and Grzybowski A. (2016) The role of the reactive oxygen species and oxidative stress in the pathomechanism of the age-related ocular diseases and other pathologies of the anterior and posterior eye segments in adults. Oxid. Med. Cell Longev. 2016, 3164734 10.1155/2016/3164734 26881021 PMC4736974

[9] Schertl P., Volk J., Perduns R., Adam K., Leyhausen G., Bakopoulou A. et al. (2019) Impaired angiogenic differentiation of dental pulp stem cells during exposure to the resinous monomer triethylene glycol dimethacrylate. Dent. Mater. 35, 144–155 10.1016/j.dental.2018.11.006 30502225

[10] Bakopoulou A., Papadopoulos T. and Garefis P. (2009) Molecular toxicology of substances released from resin-based dental restorative materials. Int. J. Mol. Sci. 10, 3861–3899 10.3390/ijms10093861 19865523 PMC2769064

[11] Schweikl H., Altmannberger I., Hanser N., Hiller K.A., Bolay C., Brockhoff G. et al. (2005) The effect of triethylene glycol dimethacrylate on the cell cycle of mammalian cells. Biomaterials 26, 4111–4118 10.1016/j.biomaterials.2004.10.026 15664638

[12] Tigani E.K., Skrtic D., Valerio M.S. and Kaufman G. (2019) Assessing the effect of triethyleneglycol dimethacrylate on tissue repair in 3D organotypic cultures. J. Appl. Toxicol. 39, 247–259 10.1002/jat.3714 30229966

[13] Noda M., Wataha J.C., Kaga M., Lockwood P.E., Volkmann K.R. and Sano H. (2002) Components of dentinal adhesives modulate heat shock protein 72 expression in heat-stressed THP-1 human monocytes at sublethal concentrations. J. Dent. Res. 81, 265–269 10.1177/154405910208100408 12097311

[14] Nocca G., Calla C., Martorana G.E., Cicillini L., Rengo S., Lupi A. et al. (2014) Effects of dental methacrylates on oxygen consumption and redox status of human pulp cells. Biomed. Res. Int. 2014, 956579 10.1155/2014/956579 24693541 PMC3944953

[15] Schweikl H., Hiller K.A., Eckhardt A., Bolay C., Spagnuolo G., Stempfl T. et al. (2008) Differential gene expression involved in oxidative stress response caused by triethylene glycol dimethacrylate. Biomaterials 29, 1377–1387 10.1016/j.biomaterials.2007.11.049 18164055

[16] Brugarolas J., Lei K., Hurley R.L., Manning B.D., Reiling J.H., Hafen E. et al. (2004) Regulation of mTOR function in response to hypoxia by REDD1 and the TSC1/TSC2 tumor suppressor complex. Genes Dev. 18, 2893–2904 10.1101/gad.1256804 15545625 PMC534650

[17] Tirado-Hurtado I., Fajardo W. and Pinto J.A. (2018) DNA damage inducible transcript 4 gene: the switch of the metabolism as potential target in cancer. Front. Oncol. 8, 106 10.3389/fonc.2018.00106 29707520 PMC5906527

[18] Gharibi B., Ghuman M. and Hughes F.J. (2016) DDIT4 regulates mesenchymal stem cell fate by mediating between HIF1alpha and mTOR signalling. Sci. Rep. 6, 36889 10.1038/srep36889 27876894 PMC5120275

[19] Saxton R.A. and Sabatini D.M. (2017) mTOR Signaling in growth, metabolism, and disease. Cell 169, 361–371 10.1016/j.cell.2017.03.035 28388417

[20] Laplante M. and Sabatini D.M. (2012) mTOR signaling in growth control and disease. Cell 149, 274–293 10.1016/j.cell.2012.03.017 22500797 PMC3331679

[21] Mills J.R., Hippo Y., Robert F., Chen S.M., Malina A., Lin C.J. et al. (2008) mTORC1 promotes survival through translational control of Mcl-1. Proc. Natl. Acad. Sci. U.S.A. 105, 10853–10858 10.1073/pnas.0804821105 18664580 PMC2504845

[22] Park Y., Reyna-Neyra A., Philippe L. and Thoreen C.C. (2017) mTORC1 balances cellular amino acid supply with demand for protein synthesis through post-transcriptional control of ATF4. Cell Rep. 19, 1083–1090 10.1016/j.celrep.2017.04.042 28494858 PMC5811220

[23] Akhmanova M., Osidak E., Domogatsky S., Rodin S. and Domogatskaya A. (2015) Physical, spatial, and molecular aspects of extracellular matrix of in vivo niches and artificial scaffolds relevant to stem cells research. Stem Cells Int. 2015, 167025 10.1155/2015/167025 26351461 PMC4553184

[24] Huang N.F., Patlolla B., Abilez O., Sharma H., Rajadas J., Beygui R.E. et al. (2010) A matrix micropatterning platform for cell localization and stem cell fate determination. Acta Biomater. 6, 4614–4621 10.1016/j.actbio.2010.06.033 20601236 PMC2957527

[25] Chowdhury F., Li Y., Poh Y.C., Yokohama-Tamaki T., Wang N. and Tanaka T.S. (2010) Soft substrates promote homogeneous self-renewal of embryonic stem cells via downregulating cell-matrix tractions. PLoS ONE 5, e15655 10.1371/journal.pone.0015655 21179449 PMC3001487

[26] Arakaki M., Ishikawa M., Nakamura T., Iwamoto T., Yamada A., Fukumoto E. et al. (2012) Role of epithelial-stem cell interactions during dental cell differentiation. J. Biol. Chem. 287, 10590–10601 10.1074/jbc.M111.285874 22298769 PMC3323010

[27] Yoshizaki K. and Yamada Y. (2013) Gene evolution and functions of extracellular matrix proteins in teeth. Orthod. Waves 72, 1–10 10.1016/j.odw.2013.01.040 23539364 PMC3607546

[28] Sawada T. and Inoue S. (1998) Basement membrane-like structures occurring on the surface of dental papilla mesenchymal cells during odontogenesis in the monkey *Macaca fuscata*. Eur. J. Oral Sci. 106, 126–131 10.1111/j.1600-0722.1998.tb02164.x 9541214

[29] Kawashima N. and Okiji T. (2016) Odontoblasts: specialized hard-tissue-forming cells in the dentin-pulp complex. Congenit. Anom. (Kyoto) 56, 144–153 10.1111/cga.12169 27131345

[30] Yuasa K., Fukumoto S., Kamasaki Y., Yamada A., Fukumoto E., Kanaoka K. et al. (2004) Laminin alpha2 is essential for odontoblast differentiation regulating dentin sialoprotein expression. J. Biol. Chem. 279, 10286–10292 10.1074/jbc.M310013200 14681233

[31] Ruch J.V., Lesot H. and Begue-Kirn C. (1995) Odontoblast differentiation. Int. J. Dev. Biol. 39, 51–68 7626422

[32] Sloan A.J. and Smith A.J. (2007) Stem cells and the dental pulp: potential roles in dentine regeneration and repair. Oral Dis. 13, 151–157 10.1111/j.1601-0825.2006.01346.x 17305615

[33] Farges J.C., Alliot-Licht B., Renard E., Ducret M., Gaudin A., Smith A.J. et al. (2015) Dental pulp defence and repair mechanisms in dental caries. Mediators Inflamm. 2015, 230251 10.1155/2015/230251 26538821 PMC4619960

[34] Rombouts C., Jeanneau C., Bakopoulou A. and About I. (2016) Dental pulp stem cell recruitment signals within injured dental pulp tissue. Dent. J. (Basel) 4, 29563450 10.3390/dj4020008 PMC5851269

[35] Hilkens P., Gervois P., Fanton Y., Vanormelingen J., Martens W., Struys T. et al. (2013) Effect of isolation methodology on stem cell properties and multilineage differentiation potential of human dental pulp stem cells. Cell Tissue Res. 353, 65–78 10.1007/s00441-013-1630-x 23715720

[36] Urraca N., Memon R., El-Iyachi I., Goorha S., Valdez C., Tran Q.T. et al. (2015) Characterization of neurons from immortalized dental pulp stem cells for the study of neurogenetic disorders. Stem Cell Res. 15, 722–730 10.1016/j.scr.2015.11.004 26599327 PMC4698085

[37] Raj A., van den Bogaard P., Rifkin S.A., van Oudenaarden A. and Tyagi S. (2008) Imaging individual mRNA molecules using multiple singly labeled probes. Nat. Methods 5, 877–879 10.1038/nmeth.1253 18806792 PMC3126653

[38] Huang C.E., Hu F.W., Yu C.H., Tsai L.L., Lee T.H., Chou M.Y. et al. (2014) Concurrent expression of Oct4 and Nanog maintains mesenchymal stem-like property of human dental pulp cells. Int. J. Mol. Sci. 15, 18623–18639 10.3390/ijms151018623 25322154 PMC4227236

[39] Rodas-Junco B.A., Canul-Chan M., Rojas-Herrera R.A., De-la-Pena C. and Nic-Can G.I. (2017) Stem cells from dental pulp: what epigenetics can do with your tooth. Front Physiol. 8, 999 10.3389/fphys.2017.00999 29270128 PMC5724083

[40] Maxim M.A., Soritau O., Baciut M., Bran S. and Baciut G. (2015) The role of dental stem cells in regeneration. Clujul Med. 88, 479–482 26733745 10.15386/cjmed-475 PMC4689240

[41] Gandia C., Arminan A., Garcia-Verdugo J.M., Lledo E., Ruiz A., Minana M.D. et al. (2008) Human dental pulp stem cells improve left ventricular function, induce angiogenesis, and reduce infarct size in rats with acute myocardial infarction. Stem Cells 26, 638–645 10.1634/stemcells.2007-0484 18079433

[42] Diomede F., Rajan T.S., Gatta V., D’Aurora M., Merciaro I., Marchisio M. et al. (2017) Stemness maintenance properties in human oral stem cells after long-term passage. Stem Cells Int. 2017, 5651287 10.1155/2017/5651287 28469672 PMC5392399

[43] Modena K.C., Casas-Apayco L.C., Atta M.T., Costa C.A., Hebling J., Sipert C.R. et al. (2009) Cytotoxicity and biocompatibility of direct and indirect pulp capping materials. J. Appl. Oral Sci. 17, 544–554 10.1590/S1678-77572009000600002 20027424 PMC4327511

[44] Moharamzadeh K., Brook I.M. and Noort R.V. (2009) Biocompatibility of resin-based dental materials. Materials (Basal) 2, 514–548 10.3390/ma2020514

[45] Spagnuolo G., Galler K., Schmalz G., Cosentino C., Rengo S. and Schweikl H. (2004) Inhibition of phosphatidylinositol 3-kinase amplifies TEGDMA-induced apoptosis in primary human pulp cells. J. Dent. Res. 83, 703–707 10.1177/154405910408300909 15329376

[46] Cho S.G., Lee J.W., Heo J.S. and Kim S.Y. (2014) Gene expression change in human dental pulp cells exposed to a low-level toxic concentration of triethylene glycol dimethacrylate: an RNA-seq analysis. Basic Clin. Pharmacol. Toxicol. 115, 282–290 10.1111/bcpt.12197 24438040

[47] Oncel Torun Z., Torun D., Baykal B., Oztuna A., Yesildal F. and Avcu F. (2017) Effects of triethylene glycol dimethacrylate (TEGDMA) on the odontoclastic differentiation ability of human dental pulp cells. J. Appl. Oral Sci. 25, 631–640 10.1590/1678-7757-2016-0626 29211284 PMC5701533

[48] Wolff N.C., McKay R.M. and Brugarolas J. (2014) REDD1/DDIT4-independent mTORC1 inhibition and apoptosis by glucocorticoids in thymocytes. Mol. Cancer Res. 12, 867–877 10.1158/1541-7786.MCR-13-0625 24615339 PMC4260655

[49] Jhanwar-Uniyal M., Wainwright J.V., Mohan A.L., Tobias M.E., Murali R., Gandhi C.D. et al. (2019) Diverse signaling mechanisms of mTOR complexes: mTORC1 and mTORC2 in forming a formidable relationship. Adv. Biol. Regul. 72, 51–62 10.1016/j.jbior.2019.03.003 31010692

[50] Kim E.Y., Kim A., Kim S.K., Kim H.J., Chang J., Ahn C.M. et al. (2014) Inhibition of mTORC1 induces loss of E-cadherin through AKT/GSK-3beta signaling-mediated upregulation of E-cadherin repressor complexes in non-small cell lung cancer cells. Respir. Res. 15, 26 10.1186/1465-9921-15-26 24571487 PMC3941688

[51] Wei G., Wang L., Dong D., Teng Z., Shi Z., Wang K. et al. (2018) Promotion of cell growth and adhesion of a peptide hydrogel scaffold via mTOR/cadherin signaling. J. Cell. Physiol. 233, 822–829 10.1002/jcp.25864 28213972

[52] Meng D., Frank A.R. and Jewell J.L. (2018) mTOR signaling in stem and progenitor cells. Development 145, 10.1242/dev.152595 PMC5825873 29311260

[53] Martin S.K., Fitter S., Dutta A.K., Matthews M.P., Walkley C.R., Hall M.N. et al. (2015) Brief report: the differential roles of mTORC1 and mTORC2 in mesenchymal stem cell differentiation. Stem Cells 33, 1359–1365 10.1002/stem.1931 25537496

[54] Xian L., Wu X., Pang L., Lou M., Rosen C.J., Qiu T. et al. (2012) Matrix IGF-1 maintains bone mass by activation of mTOR in mesenchymal stem cells. Nat. Med. 18, 1095–1101 10.1038/nm.2793 22729283 PMC3438316

[55] Fong H., Hohenstein K.A. and Donovan P.J. (2008) Regulation of self-renewal and pluripotency by Sox2 in human embryonic stem cells. Stem Cells 26, 1931–1938 10.1634/stemcells.2007-1002 18388306

[56] Strebinger D., Deluz C., Friman E.T., Govindan S., Alber A.B. and Suter D.M. (2019) Endogenous fluctuations of OCT4 and SOX2 bias pluripotent cell fate decisions. Mol. Syst. Biol. 15, e9002 10.15252/msb.20199002 31556488 PMC6759502

[57] Nuti N., Corallo C., Chan B.M., Ferrari M. and Gerami-Naini B. (2016) Multipotent differentiation of human dental pulp stem cells: a literature review. Stem Cell Rev. Rep. 12, 511–523 10.1007/s12015-016-9661-9 27240827

[58] Tsai C.C., Su P.F., Huang Y.F., Yew T.L. and Hung S.C. (2012) Oct4 and Nanog directly regulate Dnmt1 to maintain self-renewal and undifferentiated state in mesenchymal stem cells. Mol. Cell 47, 169–182 10.1016/j.molcel.2012.06.020 22795133

[59] Bollini S., Gentili C., Tasso R. and Cancedda R. (2013) The regenerative role of the fetal and adult stem cell secretome. J. Clin. Med. 2, 302–327 10.3390/jcm2040302 26237150 PMC4470151

[60] Batouli S., Miura M., Brahim J., Tsutsui T.W., Fisher L.W., Gronthos S. et al. (2003) Comparison of stem-cell-mediated osteogenesis and dentinogenesis. J. Dent. Res. 82, 976–981 10.1177/154405910308201208 14630898

