# Supplementary material for: The self-renewal dental pulp stem cell microtissues challenged by a toxic dental monomer
Source: Biosci Rep. 2020 Jun 18;40(6):BSR20200210. doi: 10.1042/BSR20200210 (PMC7303350; doi:10.1042/BSR20200210)
Supplement: Supplementary Table S1 [file BSR-2020-0210_supp.pdf]

Table 1

Probe name	Sequence (5' to 3')
H_DDIT4_1	cgtgccaaccaccgagag
H_DDIT4_2	cttgaatgggtgtgcgaa
H_DDIT4_3	aagacaagtgcgtcctgc
H_DDIT4_4	cgcggtcagcgagaactg
H_DDIT4_5	agcgtagaagccgcagct
H_DDIT4_6	tgatgaactcagagtgcc
H_DDIT4_7	acagacgccagggcgttt
H_DDIT4_8	gacgacgagaagcggtcc
H_DDIT4_9	agggcgaagaggaggtgg
H_DDIT4_10	gatctggggtgggagttc
H_DDIT4_11	tcgtggagcggtcaaacc
H_DDIT4_12	caggtaagccgtgtcttc
H_DDIT4_13	agtcgggcaacgacaccc
H_DDIT4_14	agggtcactgagcagctc
H_DDIT4_15	gcacacaagtgttcatcc
H_DDIT4_16	taggcatcagcaggcgcg
H_DDIT4_17	cacctggcttaccaactg
H_DDIT4_18	cacgcagacgtccagcag
H_DDIT4_19	acgctgtggcagctcttg
H_DDIT4_20	cacgagggtcagctggaa
H_DDIT4_21	cagagtcgtgagtccagg
H_DDIT4_22	taaacagcccctggatct
H_DDIT4_23	gatgactcggaagccagt
H_DDIT4_24	cgagctgtacagcttctt
H_DDIT4_25	tcctcaatgagcagctgt
H_DDIT4_26	ccctcaggttgaagttca
H_DDIT4_27	cagtcgtctctgtcttgg
H_DDIT4_28	gaaacactattcccccac
H_DDIT4_29	cacccgcacacaactcaa
H_DDIT4_30	actgaggggtatgtgtcc
H_DDIT4_31	ccctaagcctttgtttca
H_DDIT4_32	ccactgttaactgtcagt
H_DDIT4_33	agctgctctctggatgtc
H_DDIT4_34	tgctctgaggagcacgtg
H_DDIT4_35	cccttcagaccagtaaga
H_DDIT4_36	gtagtgatgctccgatca
H_DDIT4_37	tagctgcctacaacaggt
H_DDIT4_38	cccttcctactcttacat
H_DDIT4_39	caagtgatccctgacacc
H_DDIT4_40	gccaggtgtaatttttca
H_DDIT4_41	gggggaaggcttaaacgc
H_DDIT4_42	tcaactctgcagtacacg
H_DDIT4_43	gaagacccagatggcact
